# Contribution of Intrinsic Lactate to Maintenance of Seizure Activity in Neocortical Slices from Patients with Temporal Lobe Epilepsy and in Rat Entorhinal Cortex

**DOI:** 10.3390/ijms18091835

**Published:** 2017-08-23

**Authors:** Eskedar Ayele Angamo, Rizwan ul Haq, Jörg Rösner, Siegrun Gabriel, Zoltán Gerevich, Uwe Heinemann, Richard Kovács

**Affiliations:** 1 Neuroscience Research Center, Charité—Universitätsmedizin Berlin, corporate member of Freie Universität Berlin, Humboldt-Universität zu Berlin, and Berlin Institute of Health, Berlin, Charitéplatz 1, 10117 Berlin, Germany; eskedar.angamo@charite.de (E.A.A.); joerg.roesner@charite.de (J.R.); siegrun.gabriel@charite.de (S.G.); mozsafa@gmail.com (U.H.); 2 Institute for Neurophysiology, Charité—Universitätsmedizin Berlin, corporate member of Freie Universität Berlin, Humboldt-Universität zu Berlin, and Berlin Institute of Health, Berlin, Charitéplatz 1, 10117 Berlin, Germany; zoltan.gerevich@charite.de; 3 Department of Pharmaceutical Sciences, Abbottabad University of Sciences and Technology, Abbottabad 22500, Pakistan; rizwanhej@gmail.com

**Keywords:** lactate, monocarboxylate transporter inhibitors, seizure, interictal activity, mesial temporal lobe epilepsy, adenosine

## Abstract

Neuronal lactate uptake supports energy metabolism associated with synaptic signaling and recovery of extracellular ion gradients following neuronal activation. Altered expression of the monocarboxylate transporters (MCT) in temporal lobe epilepsy (TLE) hampers lactate removal into the bloodstream. The resulting increase in parenchymal lactate levels might exert both, anti- and pro-ictogen effects, by causing acidosis and by supplementing energy metabolism, respectively. Hence, we assessed the contribution of lactate to the maintenance of transmembrane potassium gradients, synaptic signaling and pathological network activity in chronic epileptic human tissue. Stimulus induced and spontaneous field potentials and extracellular potassium concentration changes (∆[K^+^]_O_) were recorded in parallel with tissue pO_2_ and pH in slices from TLE patients while blocking MCTs by α-cyano-4-hydroxycinnamic acid (4-CIN) or d-lactate. Intrinsic lactate contributed to the oxidative energy metabolism in chronic epileptic tissue as revealed by the changes in pO_2_ following blockade of lactate uptake. However, unlike the results in rat hippocampus, ∆[K^+^]_O_ recovery kinetics and field potential amplitude did not depend on the presence of lactate. Remarkably, inhibition of lactate uptake exerted pH-independent anti-seizure effects both in healthy rat and chronic epileptic tissue and this effect was partly mediated via adenosine 1 receptor activation following decreased oxidative metabolism.

## 1. Introduction

Under physiological conditions, brain function predominantly relies on oxidative metabolism of glucose [1]. During extensive neuronal activation, glial glycogen stores are mobilized and surplus lactate from the glycolysis is released via monocarboxylate transporters (MCT), hemichannels or ion channels into the extracellular space [2,3,4] generating a lactate gradient between astrocytes, neurons and blood vessels [5]. Due to the delicate distribution pattern of MCTs on different cell types and subcellular compartments, individual components of synaptic signaling might differentially rely on ATP derived from the oxidative metabolism of lactate [6]. Although the general validity of astrocytic neuronal lactate shuttle (ANLS) [7] has been a matter of debate [8], a subtle but important role for lactate can be found in the presence of glucose in neurovascular signaling [9], synaptic transmission and plasticity [10,11,12,13,14]. The low affinity isoform MCT1 is expressed in astrocytes, endothelial cells of microvessels and on oligodendrocytes [6]. Lactate release mediated by oligodendricytic MCT1 was shown to be critical for maintenance of axonal functions [15]. MCT4 is the major MCT isoform present on astrocytes in several brain structures, including the hippocampus. The density of MCT4 appears to be highest in the area of the astrocytic endfeet enwrapping the cerebrovasculature [16,17], suggesting that MCT4 facilitates the removal of lactate into the circulation in order to prevent lactic acidosis. Although MCT2 immunoreactivity has also been found on perivascular endfeet [18,19,20], its high affinity for lactate (Km: 0.74 mM) and its preferential localization on dendritic post-synaptic densities [16,21] implicates that this isoform might be responsible for the neuronal uptake and for the metabolic support of synaptic signaling [13].

We have recently shown that intrinsic lactate supports synaptic signaling in rat hippocampal slices and is critical for extracellular ion equilibration time course following neuronal activation. Inhibition of the MCT2 mediated lactate transport by α-cyano-4-hydroxycynamate (4-CIN) led to a decrease in postsynaptic action potential generation, and this effect was dependent on the activation of K_ATP_ channels. A significant contribution of lactate to oxidative energy metabolism was evidenced by the decrease in the baseline and stimulus induced changes in pO_2_ as well as by the drift in the ratio of reduced and oxidized flavin adenine dinucleotide (FADH_2_/FAD^+^) [22].

Acute provoked epileptic seizures are associated with massive increases of glucose metabolism in order to meet the energy demand for restoration of pathologically altered transmembrane ion gradients [23]. The concomitant increases in parenchymal lactate levels could be both proconvulsive by supporting energy metabolism or anticonvulsive due to the acidotic drift in tissue pH. Interictal extracellular lactate concentration was found to be elevated in mesial temporal lobe epilepsy (mTLE) patients when compared between the epileptogenic and the non-epileptogenic hippocampus [24] indicating serious alterations in the lactate removal mechanisms. Indeed, the expression pattern of several MCTs is disturbed, MCT1 was lost on microvessels and upregulated on astrocytes in the neuropil of the hippocampal formation both in human mTLE samples and in animal models of mTLE [25,26], while cortical MCT4 expression levels were significantly lowered in mTLE patients and also in pilocarpine model of epilepsy [27].

Here, we tested the hypothesis that the contribution of intrinsic lactate to energy metabolism associated with induced stimulus and epileptiform network activity are altered in chronic epileptic tissue. In subsequent experiments, we investigated whether the effect of lactate uptake inhibitors on network activity is exclusively mediated by the change in the extracellular pH or the activation of adenosine 1 (A_1_) receptors following metabolic restriction.

## 2. Results

### 2.1. α-cyano-4-hydroxycinnamic acid (4-CIN) Decreased Stimulus Induced pO_2_ in Neocortical Slices from Patients with mesial Temporal Lobe Epilepsy (mTLE)

**Figure 1:**
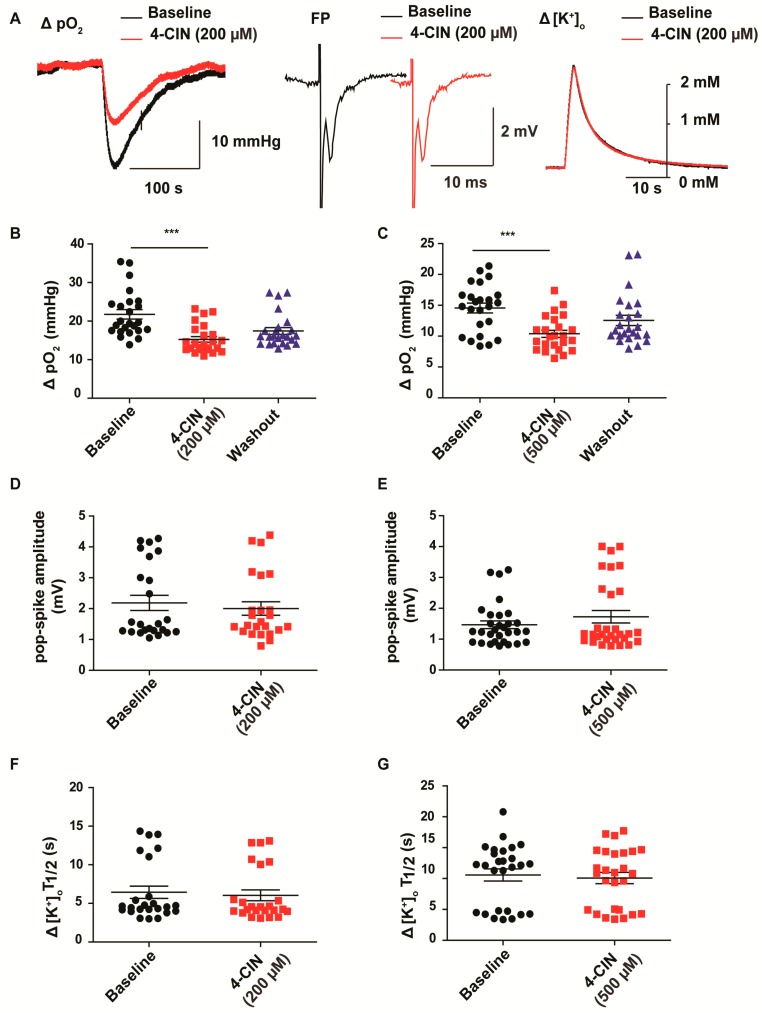
Effects of monocarboxylate transporter (MCT) inhibition by α-cyano-4-hydroxycinnamic acid (4-CIN) on stimulus induced extracellular tissue oxygen changes (ΔpO_2_), field potential responses (FP), amplitude and recovery kinetics of extracellular K^+^ concentration changes (Δ[K^+^]_O_). Despite the clear effect on the stimulus induced pO_2_ changes neither recovery kinetics of Δ[K^+^]_O_ nor field potential amplitude were affected by 4-CIN. (**A**) Sample traces of ΔpO_2_ (left), field potential transients (middle) and Δ[K^+^]_O_ (right) in the presence and the absence of 4-CIN. Inhibition of the MCTs decreased ΔpO_2_ both at (**B**) 200 µM and (**C**) 500 µM 4-CIN concentration, (**D**,**E**) whereas it did not affect field potential amplitude and (**F**,**G**) first half recovery time of Δ[K^+^]_O_ for both concentrations, respectively; (**B**–**G**) Variables are given on the *Y*-axis, categories of treatment on the *X*-axis. *** *p* < 0.001.

We have previously shown that neuronal lactate uptake supports synaptic transmission and recovery of stimulus induced transmembrane ionic gradients in the Cornu Ammonis 3 (CA3) region of the rat hippocampus [22]. Expression pattern of monocarboxylate transporters (MCTs) is altered in chronic epileptic tissue from mTLE patients [25,28], but it is not known, how these alterations would affect the role of lactate as metabolic substrate. In the first set of experiments, we tried to reproduce the findings from healthy rat hippocampus in neocortical slices from patients with mTLE. Neuronal activation was induced by application of stimulus trains (20 Hz, 2 s) onto the white matter while recording changes in extracellular K^+^ (∆[K^+^]_O_) and oxygen tension (∆pO_2_) in the deep neocortical layers V/VI. Application of 4-CIN at a concentration which preferentially blocks lactate uptake via MCT2 (200 µM) significantly reduced stimulus induced ∆pO_2_ from 21.8 ± 1.2 to 15.3 ± 0.7 mmHg (Figure 1A,B; paired *t*-test, *p* < 0.001, *n* = 9, 5 patients) in line with the findings in rat brain slices. Baseline pO_2_ of 504.4 ± 102.7 mmHg was measured in interface chamber under 95% O_2_ and 5% CO_2_. Despite the decrease in ∆pO_2_, there was no obvious change in baseline pO_2_ suggesting that lactate contribution to the basal oxidative metabolism is negligible.

Unlike in the rat hippocampus slices, the population spike components of the field potential responses were not affected by inhibition of lactate uptake (2.2 ± 0.2 mV vs. 2.0 ± 0.2 mV in control condition and during 4-CIN application respectively; Figure 1A,D; Wilcoxon signed rank test, *p* > 0.5, *n* = 8, 5 patients). In line with the missing effect on the synaptic signaling, 4-CIN did not change the recovery kinetics of stimulus induced ∆[K^+^]_O_ (peak ∆[K^+^]_O_: 1.7 ± 0.1 mM; *n* = 10, 5 patients). Neither the peak amplitude nor the 1st half decay time, 6.4 ± 0.8 vs. 6.0 ± 0.7 s, were altered (Figure 1A,F; *n* = 10, 5 patients). Taking into account the potentially different equilibration times in rat and human brain slices, we have increased the concentration of 4-CIN to 500 µM. Although this concentration readily decreased stimulus induced ∆pO_2_ from 14.6 ± 0.8 to 10.4 ± 0.6 mmHg (Figure 1C; Wilcoxon signed rank test, *p* < 0.001, *n* = 9, 5 patients), the amplitude of the evoked field potential response and the recovery kinetics of ∆[K^+^]_O_ remained unaltered (Figure 1E,G), thereby suggesting a difference in lactate use between chronic epileptic human and rat brain slices.

### 2.2. 4-CIN Reduced Incidence of Seizure-Like Events (SLEs) in Neocortical Slices from Patients with mTLE

Ictal increases in extracellular lactate levels have been described during acute provoked seizures in otherwise healthy brain slices and also in chronic epileptic tissue [29]. Surplus lactate could serve as a substrate to cover enhanced oxidative metabolism during seizure activity, whereas the acidotic shift associated with lactate accumulation have been speculated to contribute to the termination of seizures. Epileptiform activity, resembling seizure like events (SLEs), interictal and recurrent discharges can be readily induced in resected human neocortical slices by applying a combination of the GABA_A_ receptor inhibitor bicuculline methiodide (50 µM) along with an increased K^+^ (8 mM) containing aCSF [30].

**Figure 2:**
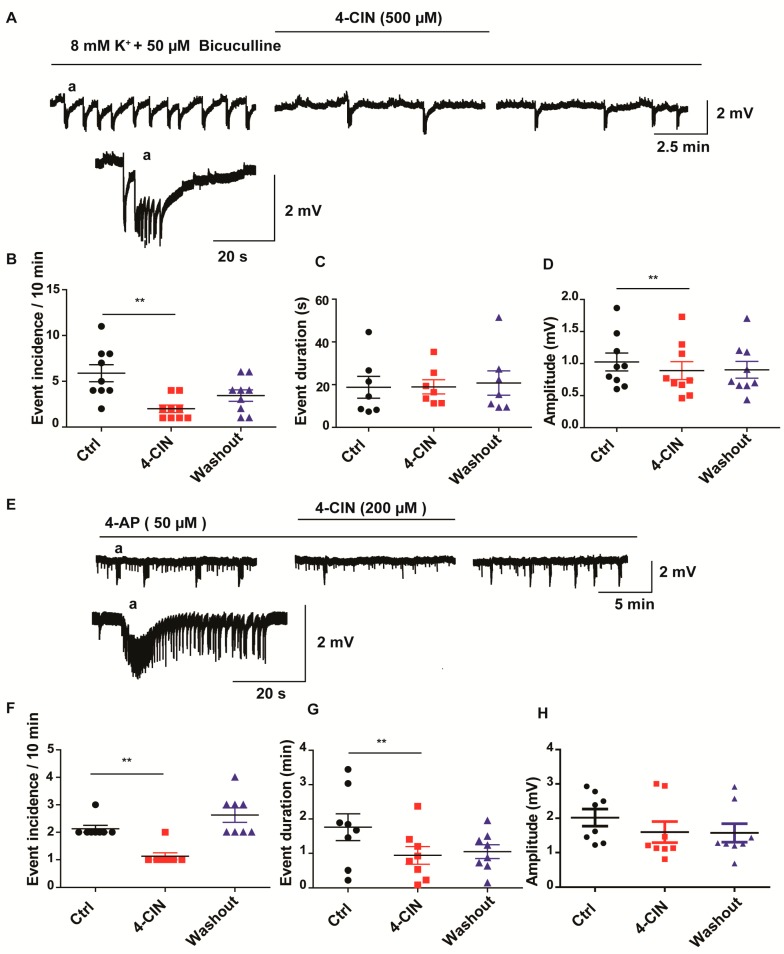
Effects of 4-CIN on spontaneous recurrent epileptiform activity induced by elevated potassium plus bicuculline in cortical slices from mesial temporal lobe epilepsy patients (mTLE) or by 4-AP in rat medial entorhinal cortex (MEC) slices. (**A**) Sample field potential trace representing recurrent SLEs during induction (**left**) 4-CIN application (**middle**) and wash out (**right**) in neocortical slices from mTLE patients, the (a) excerpt showing a single seizure like event on different time scale; (**B**) Application of 4-CIN significantly decreased incidence of SLEs (**C**) without affecting event duration (**D**) but also decreased FP amplitude; (**E**) Sample field potential trace during seizure induction (**left**) 4-CIN application (**middle**) and wash out (**right**) in MEC slices from rat, (a) the excerpt showing a single seizure like event on different time scale; (**F**) Application of 4-CIN decreased incidence and (**G**) duration of SLEs without affecting (**H**) amplitude. (**B**–**D**,**F**–**H**) Variables are given on the *Y*-axis, categories of treatment on the *X*-axis. ** *p* < 0.01. Each dot represents a single data point, black dots represent control condition (seizure induction), red dots represent 4-CIN application on top of seizure inducing drugs, and blue dots represent washout phase.

After attaining stable recurrent SLEs (first SLE appeared with a latency of 8.4 ± 4.0 min), 500 µM 4-CIN was applied for 30 min and the incidence, amplitude and duration of SLEs were evaluated. Inhibition of MCTs significantly reduced SLE incidence from 5.9 ± 0.9 to 2 ± 0.4 (Figure 2A,B; paired *t*-test, *p* < 0.01, *n* = 9 slices, 4 patients) and amplitude from 1.0 ± 0.1 to 0.9 ± 0.1 mV (Figure 2A,D; paired *t*-test, *p* <0.01, *n* = 9 slices, 4 patients) while it did not change SLE duration of the last 10 min recordings for each condition (Figure 2A,C). Notably, the effect of 4-CIN developed slowly reaching its maximum at the end of 4-CIN application. The decrease in incidence and amplitude was not due to a time dependent general decline in the propensity of the slices to generate SLEs as these parameters (incidence and amplitude) partially recovered following washout of 4-CIN to 3.4 ± 1.9 (Figure 2A,B; paired *t*-test, *p* < 0.05, *n* = 9 slices, 4 patients).

### 2.3. Lactate Uptake Inhibitors Decreased the Incidence of Pharmacologically Induced Burst Discharges and SLEs in Rat Entorhinal Cortex-Hippocampus Slices

In the next set of experiments, we determined whether the anti-seizure effect of 4-CIN was specific to chronic epileptic tissue or it represents a general consequence of MCT inhibition. Likewise, we induced SLEs in rat medial entorhinal cortex by applying the voltage gated potassium channel blocker, 4-aminopyridine (4-AP, 50 µM). Application of 4-CIN (200 µM) after establishing regular SLEs (with a latency of 21.8 ± 8.6 min) completely stopped SLEs in 2 slices out of 10 and in the rest it decreased SLE incidence from 2.1 ± 0.1 to 1.1 ± 0.1 during the last 10 min of the control and drug exposure phases (Figure 2E,F; Wilcoxon signed rank test, *p* < 0.01, *p* = 8, 4 rats) and seizure duration from 1.8 ± 0.4 to 0.9 ± 0.3 s (Figure 2E,G; paired *t*-test, *p* < 0.01, *p* = 8, 4 rats), whereas it did not affect the amplitude of SLEs (Figure 2E,H). Increasing the concentration of 4-CIN to 500 µM, as applied to human brain slices, ceased the SLEs in all slices. Notably, there was a complete recovery upon washout (*n* = 8, 4 rats).

**Figure 3:**
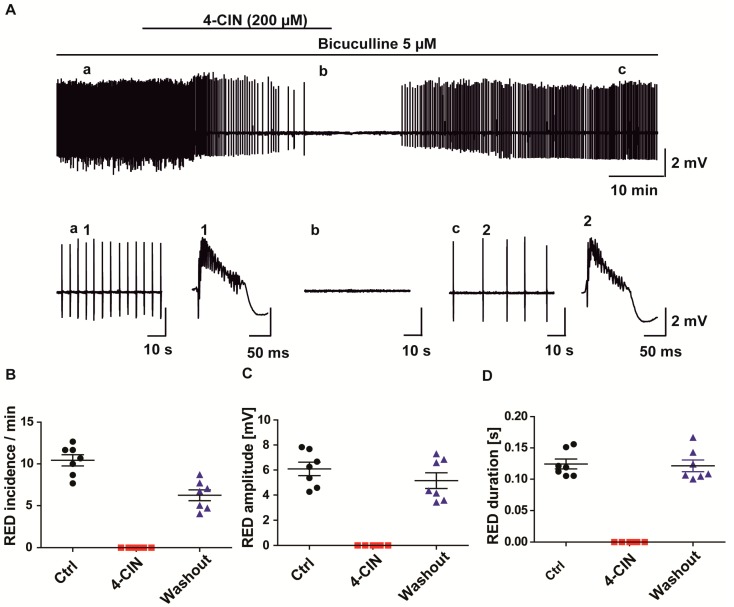
Effect of MCT inhibition by 4-CIN on recurrent epileptiform discharges (REDs) induced by pharmacological blockade of GABAA receptors. (**A**) Sample traces of bicuculline induced REDs before (**left**, **a**), during (**middle**, **b**) and after 4-CIN application (**right**, **c**). Single RED trace during baseline (1) and washout (2). 4-CIN completely stopped REDs and the effect on (**B**) incidence (**C**) amplitude and (**D**) frequency was partly reversed during washout; (**B**–**D**) Variables are given on the *Y*-axis, categories of treatment on the *X*-axis. Each dot represents a single data point, black dots represent 5 µM bicuculline application, red dots represent 200 µM 4-CIN and 5 µM bicuculline application, blue dots represent washout in 5 µM bicuculline.

In order to exclude that the observed anti-seizure effect would depend on the type of pro-convulsive treatment (bicuculline in human, 4-AP in rat), we also extended the study to bicuculline induced epileptiform activity in rat hippocampus slices. Inhibition of GABA_A_ receptors have been shown to induce recurrent epileptiform discharges (REDs) of shorter duration rather resembling interictal burst activity than of the SLEs induced by 4-AP [31]. By applying 5 µM bicuculline, we were able to induce REDs in the area CA3 with an amplitude of 6.1 ± 1.4 mV (Figure 3A,C), duration of 124 ± 7 ms (Figure 3A,D) and incidence of 10.4 ± 1.8/min (Figure 3B; *n* = 7, 3 rats). Application of 200 µM 4-CIN completely ceased REDs. During washout the incidence partially recovered while the amplitude and duration undertook complete recovery (Figure 3A–D). Thus blocking lactate uptake exerts an anti-seizure effect irrespective of the proconvulsive treatment (4-AP, bicuculline, bicuculline plus elevated potassium), species and anatomical structure (human neocortex vs. rat entorhinal cortex—hippocampus slices) as well as the type of the tissue (chronic epileptic vs. healthy control).

### 2.4. Anti-Epileptic Effect of 4-CIN Is Mediated by Adenosine through A1 Receptor But Not by Acidosis

**Figure 4:**
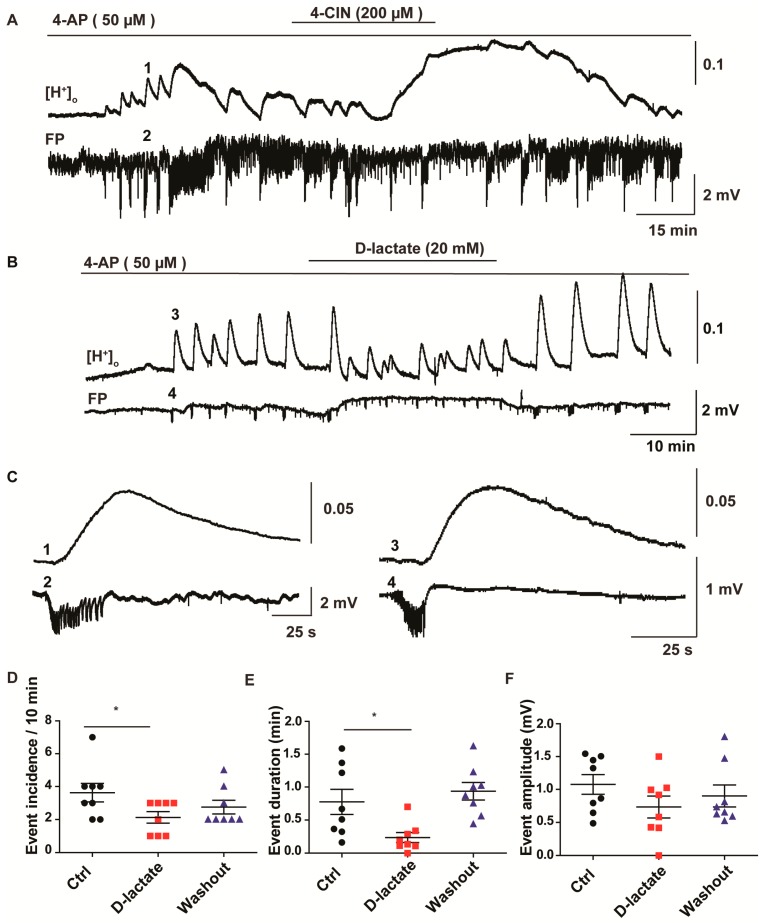
Effect of MCT inhibitors—4-CIN and d-lactate—on baseline and SLE-associated changes in extracellular pH. Extracellular H^+^ ion concentration was measured using ion sensitive electrodes; hence the displayed traces show change in H^+^ ion concentration which got converted to pH units. (**A**) Application of 4-CIN resulted in a late onset baseline acidotic shift; (**B**) In contrast, application of sodium d-lactate (20 mM, osmolality and pH set as in normal aCSF) did not induce changes in baseline pH while exerting similar inhibitory effects on 4-AP induced epileptic form activity; (**C**) Sample trace of single SLE with corresponding pH change in different time scale. Each individual SLE was associated with small acidotic shifts in both 4-CIN (1,2) and d-lactate (3,4) experiments, traces taken from the recordings in (**A**,**B**) as shown by the corresponding number; (**D**) SLE incidence and (**E**) duration (**F**) but not the amplitude were significantly decreased; (**D**–**F**) Variables are given on the *Y*-axis, categories of treatment on the *X*-axis. * *p* < 0.05. Each dot represents a single data point, black dots represent 50 µM 4-aminopyridine (4-AP) application, red dots represent 20 mM d-lactate and 50 µM 4-AP application, blue dots represent washout in 50 µM 4-AP.

In our previous paper, we have shown that 4-CIN induces extracellular acidosis due to parenchymal lactate accumulation (Figure 4A) [22]. Whether or not this acidosis could contribute to the observed anti-seizure effect was tested by using d-lactate, the non-metabolisable isoform, to inhibit lactate transport. As it competes with l-lactate on all types of MCTs, both for release and uptake, no net lactate accumulation occurs in the parenchyma and consequently no extracellular acidosis is expected (Figure 4B). In line with our previous findings, application of 4-CIN led to an acidotic shift of 0.17 ± 0.01 pH unit also in the presence of 4-AP induced epileptiform activity in rat entorhinal cortex slices [22]. Each seizure like event (SLE) was associated with a corresponding acidotic shift (Figure 4C). Although the amplitude of individual SLE-associated pH transients decreased during the 4-CIN induced pH shift, this might be simply a consequence of the simultaneous decrease of SLE duration (Figure 4A) rather than a consequence of a genuine change in metabolism. In the subsequent set of experiments d-lactate (20 mM) application following establishment of recurrent SLEs reduced SLE incidence/10 min from 3.6 ± 0.6 to 2.1 ± 0.4 (Figure 4D; paired *t*-test, *p* < 0.05, *n* = 8, 4 rats) and SLE duration from 0.78 ± 0.2 to 0.24 ± 0.1 min (Figure 4E; Wilcoxon signed rank test, *p* < 0.05, *n* = 8, 4 rats) without affecting the baseline pH. SLE amplitude was slightly decreased as well but it did not reach significance within the timeframe of the application (Figure 4F). The decrease in SLE amplitude and duration was also reflected in the decrease of the SLE-associated acidotic pH transients, despite the absence of a baseline pH change (*n* = 6, 3 rats). Similar to the anti-seizure effect of 4-CIN, the decrease in incidence and duration was reversible upon washout of d-lactate. Thus, acidosis induced by lactate accumulation cannot be a sole cause of the negative impact of MCT inhibition on seizure incidence.

**Figure 5:**
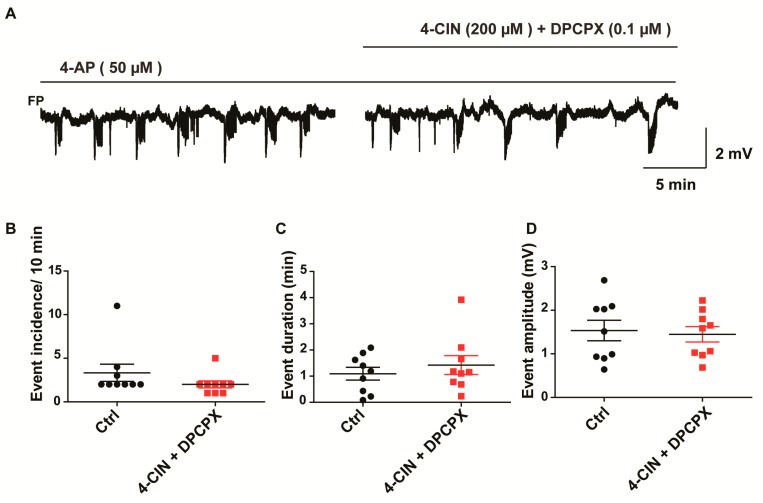
Dependence of the anti-seizure effect of MCT inhibition on the activation of A1 receptors. (**A**) Application of the A1 antagonist 8-Cyclopentyl-1,3-dipropylxanthine (DPCPX) in parallel with 4-CIN partially reversed the effect of MCT inhibitor on SLE (**B**) incidence (**C**) duration (**D**) amplitude. Variables are given on the *Y*-axis, categories of treatment on the *X*-axis. Each dot represents a single data point, black dots represent 50 µM 4-aminopyridine (4-AP) application, red dots represent 5 µM DPCPX and 50 µM 4-AP application.

The decrease in stimulus-induced ∆pO_2_ upon MCT inhibition evidenced a significant contribution of lactate to oxidative metabolism both in chronic epileptic tissue and in hippocampal slices from the rat. Conditions leading to net hydrolysis of ATP increase adenosine level which plays a neuroprotective role by suppressing excitatory neurotransmission through A1 receptors [32,33]. Seizures represent a metabolic burden leading to activation of oxidative metabolism [34,35] and increase in extracellular adenosine concentration [36], which exerts antiepileptic effects even in otherwise pharmacoresistant mTLE tissue [36]. Lactate uptake inhibitors with an effect on oxidative metabolism will presumably augment changes in extracellular adenosine. Hence, we tested whether the anti-seizure effect of 4-CIN and d-lactate is mediated by activation of the A_1_ receptor. The specific A_1_ antagonist, 8-Cyclopentyl-1,3-dipropylxanthine (DPCPX, 0.1 µM), was co-applied with 4-CIN following the establishment of recurrent SLEs in the presence of 4-AP. Although the SLE incidence was still slightly reduced from 3.3 ± 0.98 to 2.0 ± 0.4 during the concomitant application of 4-CIN and DPCPX, this change did not reach significance (Figure 5A,B; Wilcoxon signed rank test, *p* = 0.06, *n* = 9, 4 rats). Neither SLE duration (1.1 ± 0.2 vs. 1.4 ± 0.4 min; Figure 5C; Wilcoxon signed rank test, *p* = 0.2, *n* = 9, 4 rats) nor the SLE amplitude (1.5 ± 0.2 vs. 1.4 ± 0.2 mV; Figure 5D; paired *t*-test, *p* = 0.3, *n* = 9, 4 rats) were different from the control if 4-CIN and DPCPX were applied simultaneously to the aCSF. Notably, DPCPX application in the absence of 4-CIN exacerbated 4-AP induced epileptiform activity, suggesting that A_1_ receptor activation contributes to normal SLE termination and this effect might be augmented following inhibition of lactate transport. Thus, the partial reversal of the antiepileptic effect of 4-CIN by DPCPX proved that the contribution of lactate to the oxidative energy metabolism exerts a pro-seizure effect.

## 3. Discussion

The main finding of the study was that inhibition of MCT mediated lactate transport exerts an anti-seizure effect via restriction of the oxidative energy metabolism and subsequent activation of A_1_ receptors. In contrast, acidotic shift of the tissue pH was dispensable for the effect as it could be repeated by application of d-lactate, which did not result in acidosis. Remarkably, the anti-seizure effect could be observed both in chronic epileptic brain slices from TLE patients and in entorhinal cortex-hippocampus slices from rats irrespective of the type of the pro-convulsive treatment. Despite the negative effect of the inhibition of MCTs on the oxidative energy metabolism, the recovery of the stimulus induced ion transients did not depend on neuronal lactate uptake in epileptic human cortex tissue, which is in contrast to the findings in rat hippocampus slices [22].

Application of the lactate uptake inhibitor in the hippocampus decreased oxygen consumption and induced an over-oxidation of nicotinamide adenine dinucleotide (NADH) and flavin adenine dinucleotide (FADH_2_) pools, indicating that lactate is being used as oxidative energy substrate even in the presence of ample glucose [11,13,22]. Lactate derived ATP was used by ion transport mechanisms and for maintenance of postsynaptic signaling [22]. The changes in the MCT expression pattern observed in epilepsy patients as well as in animal models of epilepsy might hint to an altered use of lactate in chronic epileptic tissue [25,26,27,28]. While the increased expression of MCT1 and MCT2 in the neuropil may be the consequence of enhanced astrocytic anaerobic metabolism, the loss of MCT4 on astrocyte endfeet and of MCT1 on endothelial cells might indicate an impaired clearance into the bloodstream. These changes would favor the elevated lactate levels under conditions of enhanced neuronal activity when the brain is a net producer of lactate [37]. Taking into account the mitochondrial dysfunction in chronic epileptic tissue [38,39,40], whether this lactate is used as a substrate for oxidative metabolism in neurons is not known [24]. In our hands, MCT inhibition had a clear effect on stimulus induced pO_2_ changes in chronic epileptic tissue, pointing the relevance of lactate for oxidative energy metabolism. However, neither the amplitude of the field potentials nor the recovery kinetics of stimulus induced ion transients were altered, suggesting that these processes are not dependent on lactate derived ATP. Unfortunately, the effect of 4-CIN on the postsynaptic action potential generation might be underestimated in our model as the field potentials in the neocortical layers V/VI did not allow the clear discrimination of anti- and orthodromic population spikes and antidromic action potentials were insensitive to 4-CIN also in the rat hippocampus [22].

The most striking effect of the MCT inhibition was a decrease in the incidence of epileptiform activity up to a complete block of SLEs, irrespective of the type of the proconvulsant, i.e., 4AP, bicuculline and elevated potassium. In addition to its potential role in oxidative metabolism, an anti-seizure effect of ictal lactic acidosis has been suggested in numerous studies [41,42]. The mechanisms by which low pH may exert an anti-seizure effect might include (i) negative modulation of the *N*-methyl-d-aspartate (NMDA) receptor currents, (ii) inhibition of presynaptic voltage gated Ca^2+^ channels as well as (iii) facilitation of ecto-ATPases or increased adenosine release [41,42,43,44]. Indeed, the acidosis following 4-CIN application was associated with decreased incidence and duration of SLEs both in chronic epileptic and healthy rat brain slices. However, the anti-seizure effect was preserved also in the presence of d-lactate, which did not induce acidosis, likely due to the fact that d-lactate non-selectively inhibits both, uptake and release of lactate [13]. Thus, while acidosis is not responsible for the observed anti-seizure effect of d-lactate, it might still contribute to seizure suppression in the case of the 4-CIN treatment.

Elevated lactate by itself might alter neuronal excitability irrespective of the change in pH by acting on its G-protein coupled receptor HCA1, which reduces cellular cAMP levels [45] and inhibits action potential generation [46]. However, in our previous study the HCA1 agonist 3,5,DHBA increased orthodromic responses and stimulus associated pO_2_ changes which is not compatible with the expected inhibitory effect [22]. Therefore we would expect that—at least in rat brain slices—HCA1 activation is not likely to contribute to the inhibitory effect of parenchymal lactate accumulation.

Another possibility for an anti-seizure effect could be an increase in extracellular adenosine concentration, as a consequence of enhanced neuronal energy consumption in the presence of a partial metabolic restriction and pH dependent activation of ecto-ATPases. It has been reported previously that activity dependent basal adenosine tone modulates seizure activity [47,48] in a pH dependent manner [49] and individual seizures are associated with increased extracellular adenosine concentrations [36,50]. In general, increased adenosine levels contributed to the suppression of synaptic activity following events associated with metabolic stress such spreading depression [51], hypoxia [52], inhibition of glycolysis by 2-deoxy-d-glucose [53]. Indeed, application of the A_1_ receptor antagonist DPCPX was able to recover the effect of MCT inhibition on SLE duration and incidence. DPCPX alone exacerbated 4AP induced recurrent SLEs, suggesting that activity dependent adenosine increases might mediate seizure termination and this mechanism is augmented by restricted lactate availability. With respect to chronic epileptic tissue the relevance of A_1_ receptor modulation might be even larger as the enzyme adenosine kinase, which contributes to the termination of adenosine effects, is upregulated in astrocytes [54,55]. Indeed, application of a non-metabolisable A_1_ agonist could reverse recurrent epileptiform activity even in otherwise pharmacoresistant tissue [56].

Alternatively, substrate deprivation by MCT inhibition might also led to the activation of K_ATP_ channels, which sense the absence of lactate derived ATP, leading to hyperpolarization and suppression of postsynaptic action potential generation [22]. Whether, in our model inhibiting activation of K_ATP_ channels would be able to reverse the inhibitory effect of 4-CIN remains to be determined.

Restriction of oxidative energy metabolism by 2-deoxy-d-glucose, which also affects the lactate synthesis pathways, has been shown to have anti-epileptic effect in acute provoked seizures both in vivo and in vitro [57,58] but led to epileptogenesis in others [59]. Sada et al. 2015 has shown that inhibition of lactate dehydrogenase hyperpolarizes neurons and suppresses seizure in vivo which prompts the importance of lactate in modulating neuronal excitability [60]. Taken together these findings suggest that ictal increases in parenchymal lactate concentrations in chronic epileptic tissue are rather pro- than antiepileptic by supporting seizure associated energy metabolism.

Dietary modifications, like ketogenic diet, has been considered as an alternate treatment for certain types of drug resistant epilepsy but the mechanism of anti-epileptic action remain largely unknown [61]. One of the effects of ketogenic diet has been attributed to inhibition of glycolysis [62] which might result in a similar effect as MCT inhibition in our study. Another documented cellular adaptation in rats is an increase in the transport capacity of ketone bodies across the blood brain barrier [63], via upregulation of MCT1 expression on the brain endothelium [64]. Based on our findings, one could argue that the ketogenic diet likely restores and perhaps even increases the clearance of lactate under conditions when brain is a net producer, thereby limiting the availability of a potential energy resource.

## 4. Material and Methods

### 4.1. Slice Preparation

Animal experiments were performed on slices from 25 Wistar rats (180–250 g) in accordance with the Helsinki declaration and institutional guidelines (as approved by the State Office of Health and Social Affairs, Berlin, Germany, Lageso, T0096/02) and the animal welfare regulations of Charité. Animals were decapitated under deep anesthesia with isoflurane (3% *vol*/*vol*) and laughing gas (70% N_2_O, 30% O_2_). Horizontal hippocampal slices (400 µm thick) were prepared and transferred to an interface chamber perfused with artificial cerebrospinal fluid (aCSF) carbogenated with 95% O_2_, 5% CO_2_, at a rate of 2 mL/min. The aCSF solution is composed of NaCl (129), NaHCO_3_ (21 mM), glucose (10 mM), KCl (3 mM), NaH_2_PO_4_ (1.25 mM), CaCl_2_ (1.6 mM) and MgCl_2_ (1.8 mM) with an osmolarity of 295–305 mOsm.

The study in resected human tissue was performed after receiving written informed consent from epilepsy patients. All experiments were approved by the Ethics Committee of Charité-Universitätsmedizin Berlin on 01.11.2014 (EA2/111/14) and were in agreement with the Declaration of Helsinki. Transport, slicing and maintenance of the human tissue samples was carried out as described previously [30,56]. Immediately after surgery, the resected tissue was transferred to cold carbogenated transport solution (95% O_2_, 5% CO_2_) composed of (in mM) KCl 3, NaH_2_PO_4_ 1.25, glucose 10, sucrose 200, MgSO_4_ 2, MgCl_2_ 1.6, CaCl_2_ 1.6, and α-tocopherol 0.1 (pH 7.4, osmolality, 304 mOsmol/kg). 500 µM thick slices were prepared in transport solution and transferred to interface chamber perfused with carbogenated aCSF as specified above at a rate of 2 mL/min. Slices were left to recover for 4 h before starting an experiment.

### 4.2. Electrophysiology and Oxygen Recordings

DC coupled field potential, extracellular K^+^ ([K^+^]_O_) and pH measurements were done in layer V/VI of human neocortex and rat medial entorhinal cortex as well as in the CA3 area of the hippocampus using double barreled ion sensitive microelectrodes prepared as described in Angamo et al., 2016 [22]. For pH electrodes, the H^+^ sensitive side was filled with a solution consisting of (mM) 500 KCl, 64.7 NaH_2_PO_4_, and 85.3 Na_2_HPO_4_ (pH 7), while the reference side was filled with 500 mM KCl [22]. For K^+^ sensitive electrodes, the ion sensitive and the reference barrel were filled with 100 mM KCl and 154 NaCl solutions respectively. Then, the ion sensitive sides of K^+^ and H^+^ sensitive microelectrodes were tip filled with Potassium Ionophore I. 60031 and Hydrogen Ionophore II., Cocktail A (Fluka, Buchs, Switzerland) respectively. The sensitivity of the electrodes was tested with a tenfold calibration solution before use. Extracellular changes in K^+^ and pH were calculated using the modified Nernst equation [22]. Oxygen tension (pO_2_) was measured using the Clark-style oxygen sensor microelectrodes (tip: 10 μm; Unisense, Aarhus, Denmark) which were polarized overnight and calibrated in aCSF solution saturated with 20 and 95% O_2_. For stimulus induced responses, a bipolar platinum wire electrode was positioned in the white matter and a 20 Hz stimulus train was applied for 2 s every 4 min with an intensity giving rise of 80% of the maximal response. Data were recorded by using either a home-built differential amplifier for the ion-sensitive electrode or a polarographic amplifier (Chemical Microsensor II; Diamond General Development). Signals were digitized and recorded with a CED-1401 interface and the software Spike2 (Cambridge Electronic Design, Cambridge, UK) at 10 kHz for field potential and 1 kHz for potassium, pH and oxygen concentration.

### 4.3. Pharmacology

To inhibit the monocarboxylate transporters we used either 4-CIN (200 µM, 500 µM) or the non-metabolisable isomer sodium d-lactate (20 mM). Sodium d-lactate solution was prepared in a special aCSF with a NaCl concentration adjusted to compensate for the osmolarity change. pH of the NaHCO_3_/CO_2_ buffer system was set to 7.3 in bubbled aCSF prior to the recording. Epileptiform activity in human neocortex slices was induced by using elevated K^+^ (8 mM) aCSF solution and 50 µM bicuculline methiodide in the perfusion while in rat brain slices either 50 µM 4-aminopyridine (4-AP) or 5 µM bicuculline methiodide was added to the aCSF. The A_1_ receptor antagonist DPCPX was used at a concentration of 0.1 µM. All chemicals were bought from Sigma-Aldrich (Taufkirchen, Germany).

### 4.4. Data Analysis

The last 10 min recordings for each experimental condition (control, MCT inhibitor, and washout) were used to compare incidence, maximum amplitude including fast transients and duration of seizure like events in rat and human neocortex. For stimulus induced responses, we assessed the effect of 4-CIN on extracellular K^+^ concentration, K^+^ first half decay time, field potential responses and pO_2_. The first half decay time represents the time needed for K^+^ to reach half maximum concentration. To assess amplitude of field potential recordings in-built spike script is used; peak to point of maximum deflection is measured. The last 3 recordings (data points) during baseline and 4-CIN application were used for analysis. Data are presented as mean ± SEM, with a scatter plot where all values are displayed. Analysis was done using spike script and Graphipad Prism. The distribution of the data was tested for normality using D’Agostino and Pearson omnibus normality test; subsequently, *t*-test and Wilcoxon signed rank test were used for statistical comparison for data with normal and non-normal distribution, respectively.
